# Heat insulation effect in solar radiation of polyurethane powder coating nanocomposite

**DOI:** 10.1038/s41598-021-00181-1

**Published:** 2021-10-19

**Authors:** Ali Akbar Azemati, Seyed Saeid Rahimian Koloor, Hossain Khorasanizadeh, Michal Petrů, Ghanbar Ali Sheikhzadeh, Mahdi Safi, Behzad Shirkavand Hadavand

**Affiliations:** 1 Department of Thermo Fluids, Faculty of Mechanical Engineering, University of Kashan, Kashan, 8731753153 Iran; 2 Department of Mechanical Engineering, Abadan Branch, Islamic Azad University, Abadan, 6317836531 Iran; 3 Institute for Nanomaterials, Advanced Technologies and Innovation (CXI), Technical University of Liberec (TUL), 460 01 Liberec, Czech Republic; 4 Technical University of Liberec (TUL), 460 01 Liberec, Czech Republic; 5 Department of Color Imaging and Color Image Processing, Institute for Color Science and Technology, Tehran, 1668836471 Iran; 6 Department of Resin and Additives, Institute for Color Science and Technology, Tehran, 1668836471 Iran

**Keywords:** Engineering, Materials science, Nanoscience and technology

## Abstract

This study aims to improve polyurethane-based coating by modified zirconium oxide and aluminum oxide nanoparticles for preparing thin polymeric heat insulation coatings. In the first step, the nanoparticles were chemically modified with the silane coupling agent. Then, three different weight percent of modified nanoparticles (1, 3, and 5% w/w) were mixed with polyurethane, to prepare the nanocomposites, which were coated on metallic plate samples. Then, these plates are used to measure the radiation heat transfer coefficients, absorption coefficient in a region of short wavelengths (UV/VIS/NIR), the emissivity coefficient, and thermography of the samples in a region of long wavelengths (IR). Results showed that by adding the modified nanoparticles to the polyurethane matrix, absorption was decreased and the emissivity coefficient was increased. According to the thermography results, it was observed that the surface temperature of both samples with 3% w/w of nanoparticles had the minimum temperature compare to others. Minimum heat surface observed for 3% w/w of modified nano zirconium oxide.

## Introduction

Energy conservation has gained importance in many advanced industries as a part of eco-efficiency. In this regard, the used of advanced nanocomposites and structural coating has become one of the innovative approach in energy conservation applications^1,2^. Reinforced composites using microfibers and ceramic particles in the form of thin layer and coatings, are effectively used to enhance the mechanical features^3,4^ while reducing the emissivity coefficient and heat transfer that results in reducing the thermal energy and annual electricity consumption^5–8^, while in buildings under different climate changes. The absorption and emission of the coating material features are known to be important in surface heating, which could be controlled to produce a coating with cooling features. The cooling effect is measured by the reflection of the surface solar radiation, as well as the surface emissivity, as reflected back into the atmosphere. Such a concept was used for white materials in construction applications (e.g. roof surface) where is subjected to large solar radiation and has a high reflectance^9–12^.

A testing method has been developed to evaluate the energy performance and sustainability of innovative new products, which are known as cool colors^13^. These products were the component of selective materials (high absorption coefficient in the visible light region and high reflectance in the infrared light region), and consequently, they had both the aesthetics and cooling capabilities parameters in construction application.

Joudi^14^ studied the radiation properties of the surface, including the emissivity coefficient of interior surfaces, modeling methods, and its results on building energy performance and the thermal environment of buildings. This study indicated that surface with low emissivity can increase indoor air temperature vertical gradient, which is dependent on the time of day and outside weather conditions.

In other research by Ascione et al.^15^, a new method was implemented, in which thermo-physical properties of a building and coatings were optimized by changing the thermal resistance, capacity, and radiation properties of the surfaces exposed to radiation. In summer, the majority of cooling load faced on the building as a result of solar radiation is on the external walls of buildings. By reducing the amount of solar radiation absorbed by the external walls, the cooling load can be highly reduced. Although, the facade of the building and constructional materials used for the exterior surface is mostly decorative elements, but they play an important role in controlling the absorption of solar radiation^16^.

There reflectance properties of metal oxide nanoparticles in the infrared region (NIR) were studied by Jeevanandam and his co-works^17^. They compared reflectance ability between nanocrystalline metal oxide powder, mineral, and common macrocrystalline in the infrared region (NIR). They showed that nanocrystalline metal oxide in the infrared region (NIR) has 15–20% higher reflectance, which could be due to the coupled small crystalline particles that are produced from smaller particles according to the Kubelka–Munk theory^18^. Also, a comparison between the reflectance properties of nanocrystalline metal oxides and macrocrystalline in the near-infrared region shows that the nanocrystalline metal oxides have more reflectance^17,19^. Some researchers investigated the effect of zirconium oxide and aluminum oxide nanoparticles in ultraviolet curable coatings^20,21^, and also thermal degradation kinetics of these particles in powder coatings^22–24^. These researches intend to study the ability of nanoparticles added coating to reduce the heat transfer, where uses polyurethanes resin with high durability, resistance to the ultraviolet (UV) irradiation, and good adhesion to metallic surface features^25–27^.

As a thermal barrier, polymer coatings have to be made with specifically large thicknesses as foams or porous composites, which is the main disadvantage of such coating and limit its application. Therefore, to achieve a thin and durable coating with thermal barrier properties, a special material is required. One of the ways is to use suitable additives such as nanoparticles in the coating structure. In recent years, the use of powder coatings to coat metal products that are exposed to direct sunlight, become a common choice to prevent them from the transfer of radiant heat energy of the sun. For example, in the insulation of metal canopies and air conditioners exposed to sunlight, a thin insulating coating with a thermal insulating property is required in addition to creating a suitable appearance. Since the use of powder coatings to cover metal products has become common in recent years, and in some cases, the manufactured products are exposed to direct sunlight, it is necessary to protect the transfer of radiant thermal energy. In this regard, this study focused on the idea of using nanoparticles in powder coatings that are able to reflect sunlight, as one of the solutions to provide the coating with a proper thermal resistance in addition to a suitable low thickness.

## Materials and method

### Materials

Vinyltrimethoxysilane (VTMS) and isopropyl alcohol were provided from Merck Co. (Germany)and used for the chemical modification of nanoparticles. Formulated polyurethane powder coating resin 9016 WU18AX was prepared in Peka Chimie Co. (Iran). The nano ZrO_2_ and Al_2_O_3_ (40 nm) were purchased from US Research Nanomaterials Inc. (USA).

### Preparation of polyurethane nanocomposite

Nano ZrO_2_ and Al_2_O_3_ particles were chemically modified by a silane coupling agent^28,29^. Then, polyurethane resin and different percentages (1, 3, and 5% w/w) of modified nano ZrO_2_ and Al_2_O_3_ particles were extruded in the twin screw extruder (Yantai Donghui Powder Processing Equipment Company, China). The prepared chips were powdered and sieved to the average particle size of 55 μm. The nanocomposite was electrostatically coated on 10 × 15 cm^2^ galvanized plate and cured at 180 °C for 15 min. The composition of samples is presented in Table 1.

**Table 1:** Polyurethane power coatings nanocomposite samples.

Samples	Nano ZrO_2_ (%)	Nano Al_2_O_3_ (%)
Blank	0	0
PU-1% nano ZrO_2_	1	0
PU-3% nano ZrO_2_	3	0
PU-5% nano ZrO_2_	5	0
PU-1% nano Al_2_O_3_	0	1
PU-3% nano Al_2_O_3_	0	3
PU-5% nano Al_2_O_3_	0	5

### Instruments and test methods

The hemispherical total reflectance and emissivity were measured by emissometer/ reflectometer AZ-Technology's TEMP 2000A model (USA) in the range of long wavelengths from less than 3 µm to greater than 35 µm (infrared region) and measurement accuracy (for specular and diffuse samples) 1% of full scale for gray samples and 3% of full scale for non-gray samples. Due to the changes in the reflectance and emissivity coefficient, the coating performance is related to the wavelength in all wavelengths and then assumed as the non-gray body. This instrumentation is not limited in wavelength range due to filters, windows, or coatings, placed in the optical path. The measurements were repeated five times at different parts of each sample, according to ASTM E408 test method^30^. In long wavelengths, the absorption and emissivity coefficient for the black body was the same. Also, due to the constant temperature in the experimental test, it is assumed that thermal equilibrium is established and according to Kirchhoff's law^31^, if there is thermal equilibrium in the long wavelengths, absorption and emissivity coefficient can be considered equal for all samples^32,33^.

A large amount of solar heat gain is in the region wavelength between 0.2 and 2.5 µm (UV/Vis/NIR)^32^. Similarly, the Shimadzu spectrophotometer UV-3600 model (Japan) was used to obtain the amount of reflection coefficient of the surfaces in the same wavelengths at this region.

The wavelength accuracy in ultraviolet and visible regions is ± 0.2 nm, in near-infrared region is ± 0.8 nm, for wavelength repeat accuracy ultraviolet and visible regions is within ± 0.08 nm and for the near-infrared region is within ± 0.32 nm.

A 50 W halogen lamp and deuterium lamp (socket type) have a built-in mechanism for automatically adjusting the light-source position. The measurements were performed in the range of 282 to 393 nm at least five times at different parts of each sample according to ASTM E903-96 test method^34^.

Infrared thermography (IRT) was measured in 8–15 µm (infrared region) with infrared camera Testo, 875-2 model (Germany). According to the obtained results and given that the transmission coefficient is zero (τ = 0), the absorption coefficient can be calculated using Eq. (1)^33^:

1 $$\upalpha + \uptau + \uprho = 1$$

where α is absorption coefficient, τ is transmission coefficient and ρ is reflection coefficient.

The amount of solar irradiance on the exterior surface of the exterior is Q_r-out(i)_, which can be calculated using the equation:

2 $$Q_{r - out(i)} = \alpha q_{s} - \varepsilon \sigma (T_{s}^{4} - T_{sky}^{4} )$$

where ε is the emissivity coefficient, T_s_ is the surface temperature, q_s_ is solar incident radiation and T_sky_ is the sky temperature.

According to Eq. (2), if the emissivity coefficient is high, the amount of incident solar radiation heat flux to the exterior surfaces is reduced. This decreases the temperature of the interior surface, and therefore reduces the amount of cooling load necessary to bring the temperature of the interior space to the comfort temperature zone.

The amount of light passing through the atmosphere is not the same for all wavelengths and only in certain areas light passing is high and unlimited. Therefore, the amount of irradiance in the regions of 0.4–1.5, 1.5–2.5, 3–5, and 8–12 µm could be extraordinary and could be used for electro-optical systems. Meanwhile, only the regions of 3–5 and 8–12 µm are appropriate to make and apply passive infrared imaging system (i.e. thermography). Since, objects have significant reflectance in ambient temperature conditions, only in these two regions (3–5 and 8–12 µm) and are not absorbed by atmospheric effects.

The thermography measurement was performed outdoors and exposed to direct sunlight at noon on a sunny summer day. The samples were on a plastic platform to eliminate the effects of heat transfer between platform and samples. Data of weather conditions including temperature, relative humidity, wind speed, and amount of solar radiation are obtained from the local weather center as presented in Table 2.

**Table 2:** Environment weather conditions in the time of experiments.

Time	Air temperature (ºC)			Relative humidity (%)	Wind speed (m/s)	Maximum solar heat gain (w/m^2^)^a^
	Daily average	Daily maximum	Daily minimum			
Sunny summer day 1:00 PM	32	36	21	19	3.13	768

^a^Obtained by Carrier software.

## Results and discussion

### Morphology

**Figure 1:**
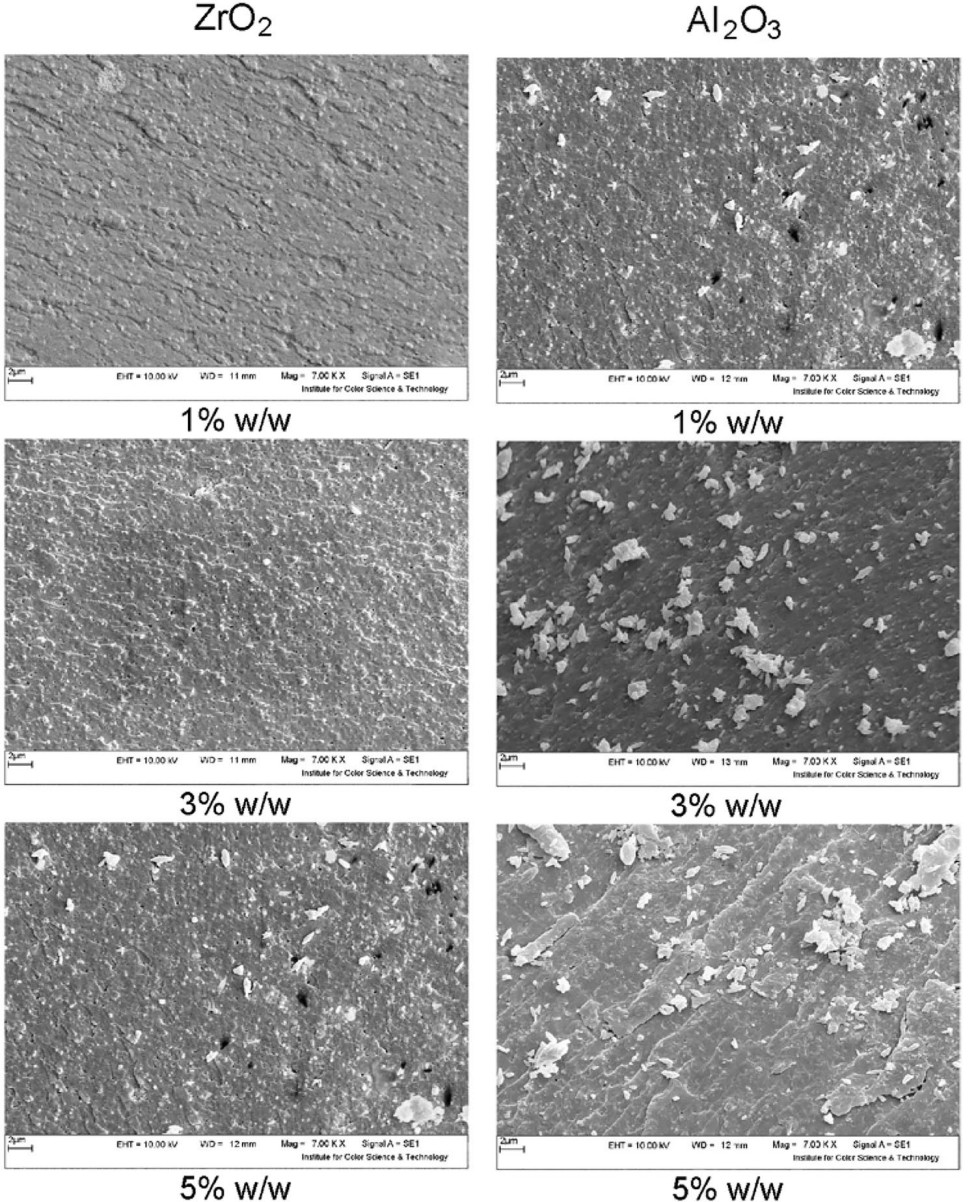
SEM images of polyurethane nanocomposite coatings with different amounts of nano ZrO_2_ and Al_2_O_3_.

The scanning electron microscope (SEM) images of polyurethane nanocomposite with different amounts of modified nano ZrO_2_ and Al_2_O_3_ particles are shown in Fig. 1. In general, a good dispersion of nanoparticles in the urethane matrix could be viewed in samples with modified nano ZrO_2_, which is the effect of modification on the dispersion of nanoparticles. The best dispersion with the suitable percentage of nanoparticles was observed in a sample with 3% w/w nano ZrO_2_ Increasing the nanoparticles to 5% w/w results in the adhesion and aggregation of the particles. It's well known that property and response of composites depends to the amount and disperse state of nano reinforcement materials^3,35,36^. The addition of nanomaterial in a certain amount could improve the properties, while the excessive addition could result in the agglomeration that causes a drop in the properties. The SEM images indicate that the reason for better absorption and emissivity coefficients for 3% w/w nanoparticles is the result of a proper dispersion of nanoparticles in a polyurethane matrix. The same results were observed in the SEM images of the modified nano Al_2_O_3_.

The dispersion in the coating is approximately the same for both particles, and the modified nanoparticles are evenly dispersed. This proper distribution is also evident in the results. However, in modified aluminum oxide nanoparticles, the aggregates of particles are higher than in zirconium oxide and larger particles are seen, which is related to the intrinsic properties of aluminum oxide and its intermolecular interactions. These gatherings are also well-distributed. As can be seen in Fig. 1, in the 5% sample of both nanoparticles, these aggregations have increased and the properties have changed.

The results of absorption and emissivity coefficients shown that coating with modified nano ZrO_2_ was acted better than modified nano Al_2_O_3_. Regardless of the differences in the nature of the particles, the microscopic images for samples with 3% w/w show that particle dispersion of modified nano ZrO_2_ is better than modified nano Al_2_O_3_, as the agglomeration of modified nano Al_2_O_3_ can be seen clearly.

### Emissivity of long wavelength

**Figure 2:**
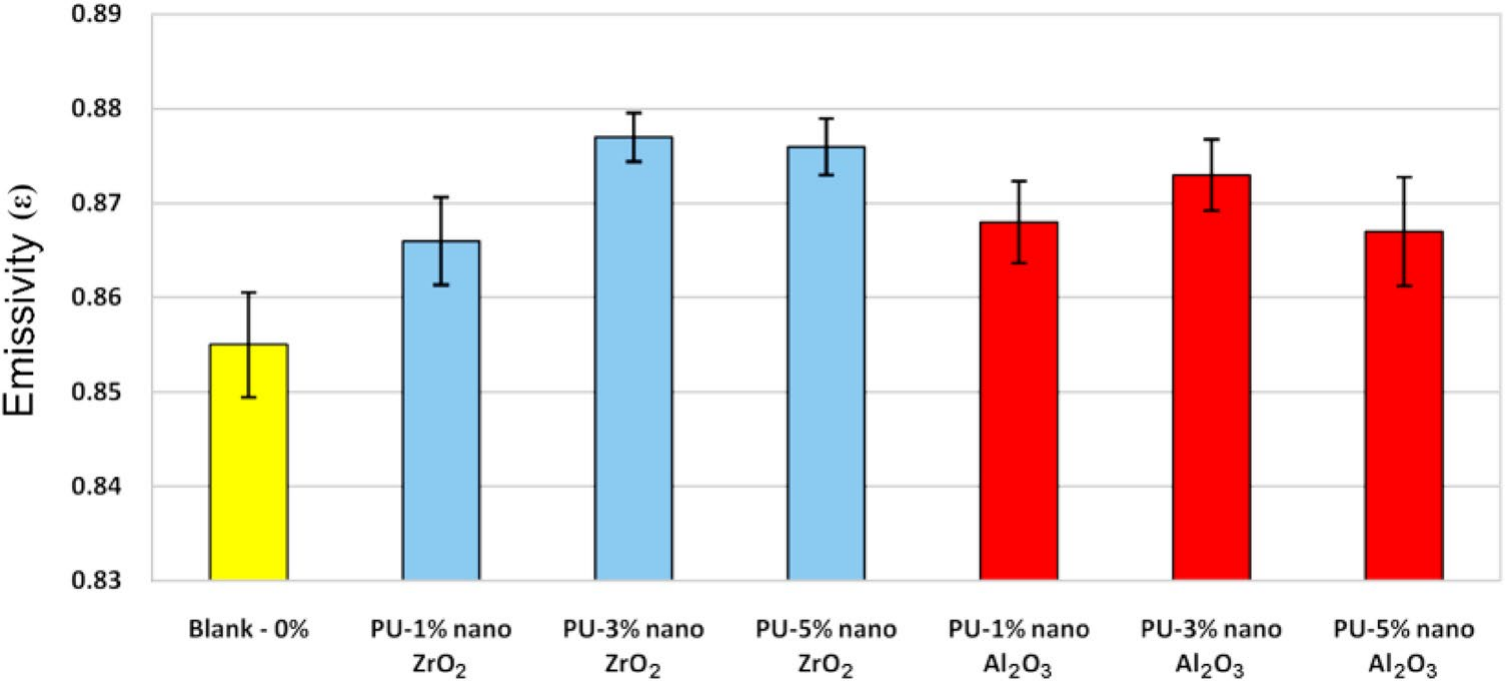
Emissivity coefficient with standard deviation for polyurethane nanocomposite coatings.

The emissivity coefficients results of the samples in the wavelengths range of 3 to 35 µm with standard deviation are presented in Fig. 2. The variance analysis of the samples was done to obtain accurate results using the data measured (minimum five times) at different parts of each sample. Results indicated that, in the wavelength of solar radiation (infrared region), the average emissivity of coatings is about 0.87. Results showed that by adding modified nano ZrO_2_ and Al_2_O_3_ to polyurethane coatings, the emissivity coefficient of coatings compare to coating without nanoparticles increased. Also, the emissivity coefficient of coatings for the sample with 3% w/w nano ZrO_2_ compare to 1 and 5% w/w of nano ZrO_2_ and 3% w/w nano Al_2_O_3_ compare to 1 and 5% w/w of nano Al_2_O_3_ are increased. It can be concluded that these nanoparticles with the optimum amount of 3% w/w have a positive role in changing the emissivity coefficient and the effect of nano ZrO_2_ are more than nano Al_2_O_3_. The reason for the higher emissivity coefficient of samples with 3% w/w nanoparticles in comparison to the samples containing 5% w/w nanoparticles is due to a better dispersion of nanoparticles in the polymeric matrix. In samples containing 5% w/w aggregation of nanoparticles has happened.

### Absorption coefficient of short wavelength

**Figure 3:**
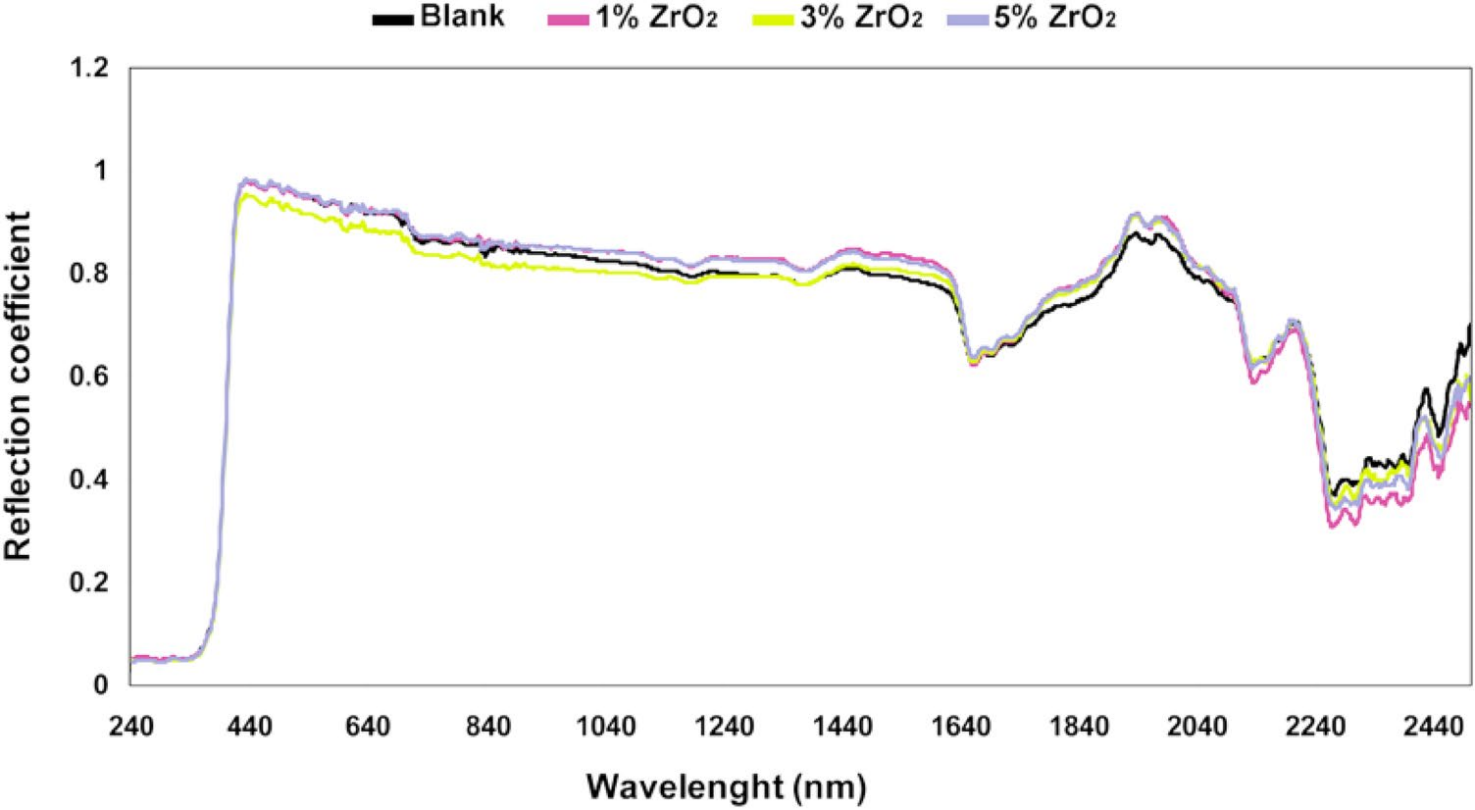
Absolute reflection coefficient of samples of nanocomposite coating for 0, 1, 3 and 5% of zirconium oxide nanoparticles in UV/VIS/NIR region.

**Figure 4:**
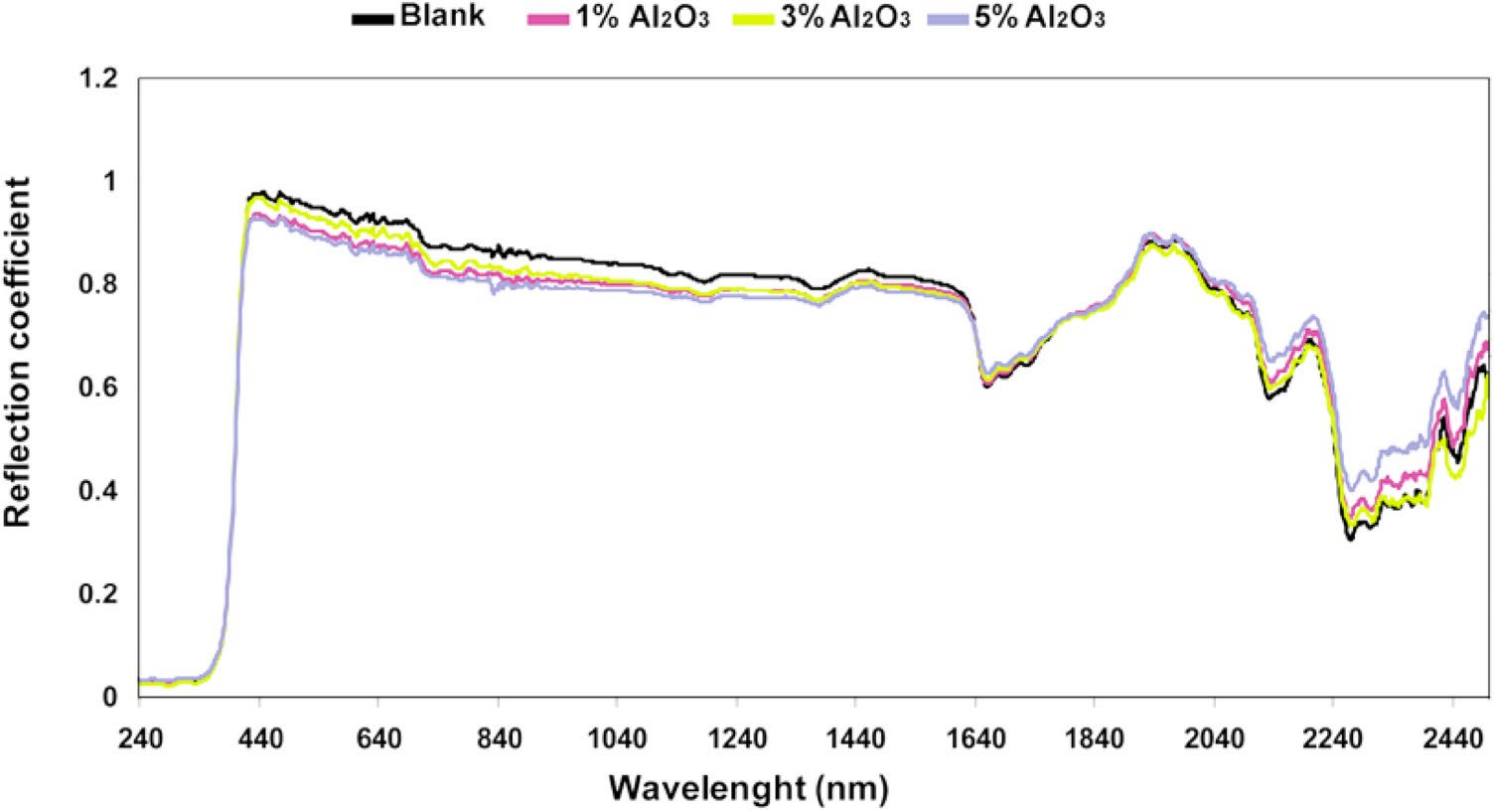
Absolute reflection coefficient of samples of nanocomposite coating for 0, 1, 3, and 5% of aluminum oxide nanoparticles in (UV/VIS/NIR) region.

The reflection coefficients of the coated samples in region wavelengths of 0.2 to 2.5 µm were measured as shown in Figs. 3 and 4. In all cases, the reflection coefficient in the UV region (about 0.4 µm) is a bit more than expected, which is encountered due to the fluorescent effect of nanoparticles in coatings, which has been reported in the literature^37^. Binder weight ratio effects and additives that adding to the polyurethane, change color and surface roughness that may help to increase reflection coefficient^38^.

**Figure 5:**
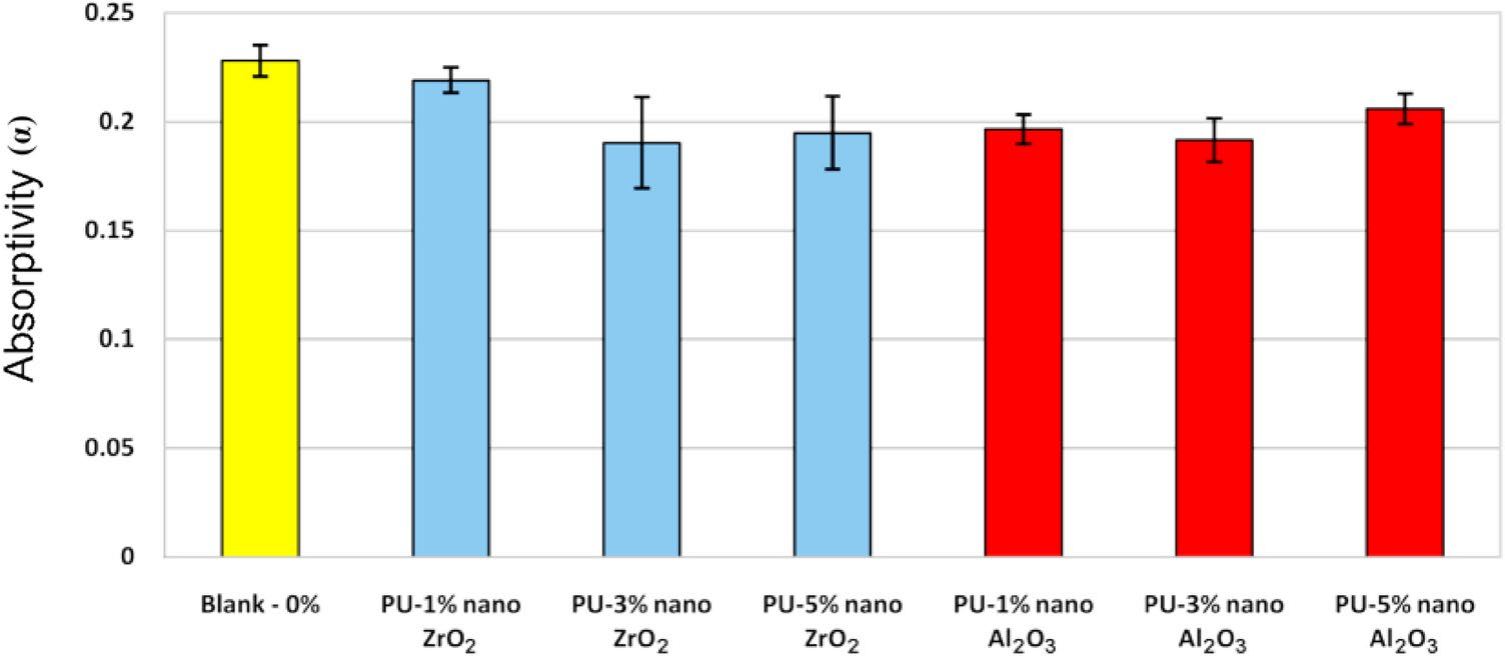
Absorption coefficient with standard deviation for polyurethane nanocomposite coatings.

Since the samples are not transmittance, the transmission coefficient is equal to zero. By integrating the results in this region, hemispherical spectral reflection coefficients were obtained, and using Eq. (1), the absorption coefficient was calculated. It is observed that in a region wavelength of UV/VIS/NIR, the absorption coefficient of the coatings is satisfied at about 0.2. The variance analysis was done to obtain accurate results using a minimum of five data measured at different parts of each sample. Figure 5 presents a comparison of absorption coefficient results of different samples with the standard deviation.

Results indicated that by adding modified nano ZrO_2_ and Al_2_O_3_to polyurethane coating, the absorption coefficient of the coating decreased in all samples compared to the coating without nanoparticles. It means that modified nano ZrO_2_ and Al_2_O_3_ have the ability to reduce absorption coefficient in coatings as a natural feature. It is worth mentioning that the same result is captured for the sample with 3% w/w nanoparticle with decreased absorption coefficient more than the other cases (the best sample for increasing the emissivity coefficient).

According to Eq. (2), it is clear that if the emissivity coefficient is high and the absorption coefficient is low, the amount of input radiation heat flux to the exterior surfaces is reduced, which, decreases the temperature of the interior surface and consequently reduces the amount of cooling load necessary to bring the space temperature to comfort temperature range.

### The infrared thermography results

**Figure 6:**
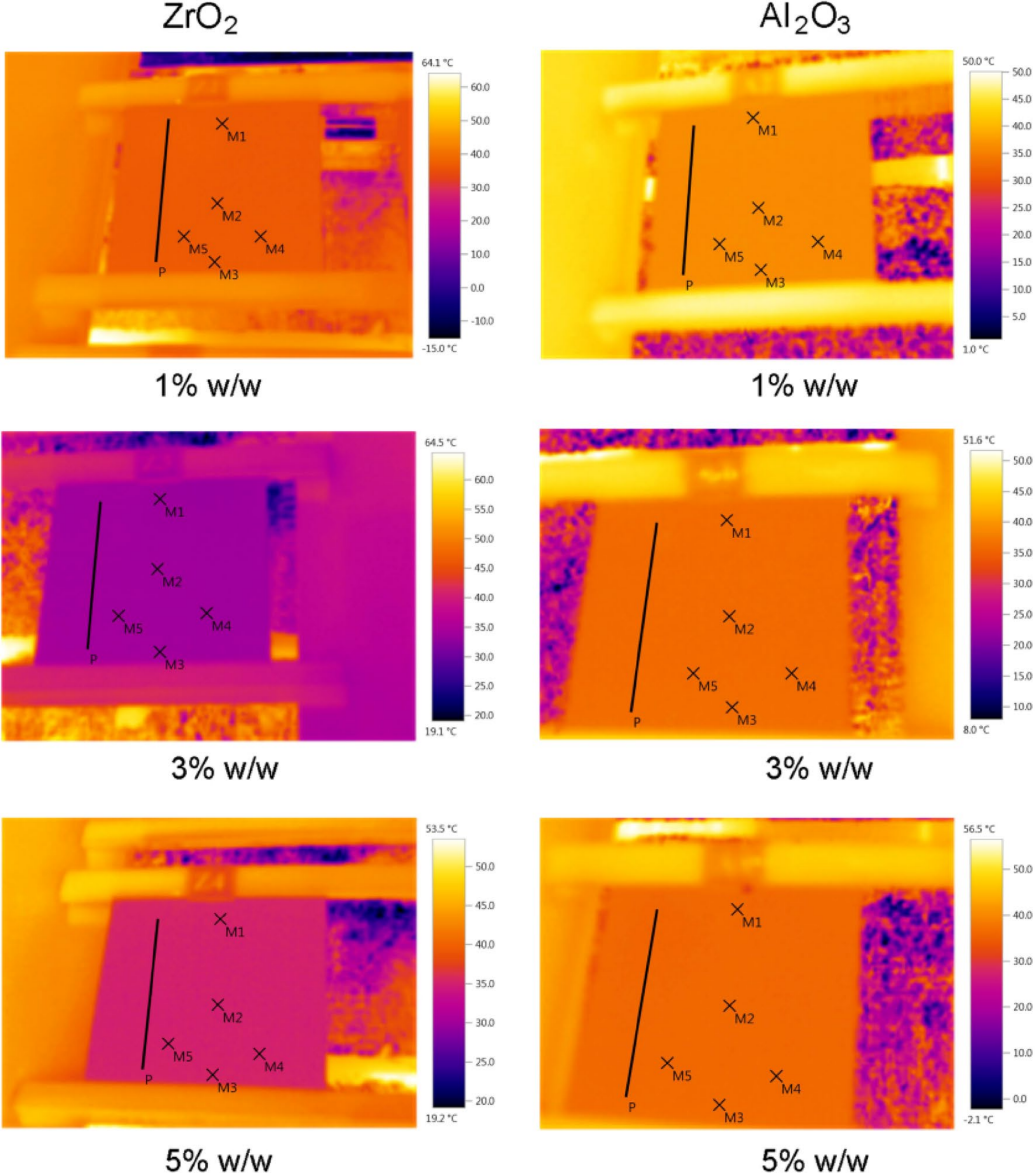
Thermography of images of polyurethane nanocomposite coatings with different amounts of nano ZrO_2_ and Al_2_O_3_.

The temperature results of infrared thermography (IRT) of polyurethane coating with different amounts of nano ZrO_2_ and Al_2_O_3_ particles are shown in Fig. 6. As indicated in this Figure, the temperature of points M1 to M5, average surface temperature, and the temperature points of the P line, were determined for each sample. It should be noted that the measurements of surfaces temperature were done according to the impact emissivity coefficient of the specimens.

It is worth mentioning that, in addition to the variation of the emissivity coefficient of the surfaces dependent on different weight percentages of nanoparticles, the temperature of the surfaces is changed as the result of the effect of different weight percentages of ZrO_2_ and Al_2_O_3_ nanoparticles.

**Figure 7:**
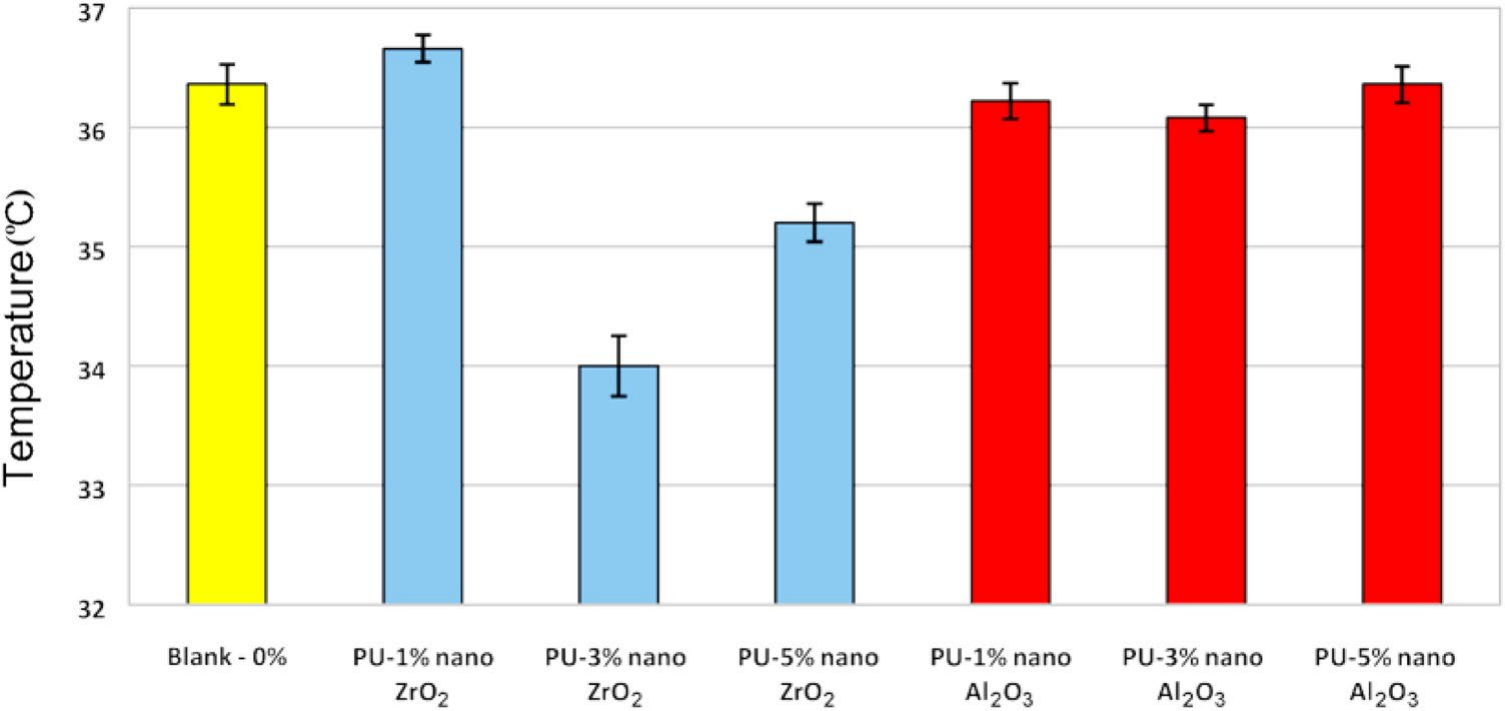
Temperature of the samples of polyurethane coating with 0, 1, 3, and 5% of nano ZrO_2_ and Al_2_O_3_.

The temperature at different points of the samples with nanocomposite coating having 0, 1, 3, and 5% of ZrO_2_ and Al_2_O_3_ nanoparticles, is shown in Fig. 7. The result indicated that the temperature at 3% is lower than the other samples, which is due to the influence of ZrO_2_ and Al_2_O_3_ nanoparticles with polyurethane matrix, dispersion quality of nanoparticles in coatings. A slight abnormal increase in temperature in the 1% zirconium oxide sample compared to the blank sample is due to the lack of unintentional unequal conditions or error at the time of measurement. But considering the errors, it is clear that it is almost equal to the blank sample.

It is worth mentioning that, the amount of flux crossing of the surface is decreasing by reducing the temperature, which causes the reduction of heat transfer reduces and the amount of energy consumption.

## Conclusion

In this study, polyurethane powder coatings nanocomposite with 1, 3, and 5% of aluminum oxide and zirconium oxide were prepared and then coated on metal plates and various tests were performed on them. Results showed that the existence of nanoparticles could have a significant effect in changing the thermal behavior of polymeric coatings. These effects depend on the nature, amount, and morphology of nanoparticles in the nanocomposite coating. Also, it is shown that by adding zirconium oxide and aluminum oxide nanoparticles to the polyurethane matrix, the emission coefficient of the coating in a region of IR was increased. The maximum emission coefficient was observed in PU/3% w/w nano ZrO_2_. In addition, the absorption coefficient of the PU/nano ZrO_2_ and Al_2_O_3_coatings in a region of UV/VIS/NIR (all cases) compared to the blank case was decreased. The minimum absorption coefficient was measured for PU/3% w/w nano ZrO_2_ case. The lowest surface temperature was determined for PU/3% w/w nano ZrO_2_ sample as proven by the thermography results.
